# Supplementary material for: Size limits the sensitivity of kinetic schemes
Source: Nat Commun. 2023 Mar 8;14:1280. doi: 10.1038/s41467-023-36705-8 (PMC9995461; doi:10.1038/s41467-023-36705-8)
Supplement: Supplementary file 1 — Supplementary Information [file 41467_2023_36705_MOESM1_ESM.pdf]

Supplementary Information

Size limits the sensitivity of kinetic schemes

Jeremy A. Owen and Jordan M. Horowitz

(Dated: February 7, 2023)

CONTENTS

Supplementary Note 1. Proof of the equilibrium equality, (10) 2

Supplementary Note 2. Application of the equilibrium equality (10) to the findings of Fukuoka et al. 4

Supplementary Note 3. Saturation of the nonequilibrium MWC bound, (11) 5

Supplementary Note 4. Exponential sensitivity by nested hysteresis 6

I. Sensitivity doubling 7

II. Iterative construction 11

III. Stabilizing extreme states to get a Hill function 12

IV. The cases $n = 2$ and $n = 3$ 13

Supplementary References 14

Supplementary References 14

Supplementary Note 1. PROOF OF THE EQUILIBRIUM EQUALITY, (10)

Here, our goal is prove that for any detailed balanced kinetic scheme, the equality

$$\frac{d \log \pi_X}{d \log x} = \left[\langle n_b \rangle_X - \langle n_b \rangle_{\overline{X}}\right](1 - \pi_X), \tag{S1}$$

holds. This is (10) from the main text. Recall the X is a set of system states, $\overline{X}$ is the set of system states not in X, and $\pi_X = \sum_{i \in X} \pi_i$.

We will now give a detailed proof of (10) directly from the principle of detailed balance. However, note that (10) can equivalently be derived using grand canonical ensemble, which says the probability of any system state in which n_b ligands are bound is proportional to $\exp(\beta\mu n_b) \sim \exp(n_b \log x)$, where $\beta\mu$ is the chemical potential of the ligand, and the proportionality "$\sim$" elides factors that do not depend on the ligand concentration, x. Differentiating the resulting expressions yields the result.

To prove (10) directly from the principle of detailed balance, we begin by expressing the steady-state probability of a state i in terms of ratios of steady-state probabilities, using

simply the normalization of probability, $\sum_j \pi_j = 1$, to get

$$\pi_i = \frac{1}{1 + \sum_{j \neq i} \frac{\pi_j}{\pi_i}}. \tag{S2}$$

For any detailed balance scheme, the ratio of the probabilities of two states i and j depends only on the ratio of transition rates along a path $i \to 1 \to 2 \to \cdots k \to j$ connecting them, as follows:

$$\frac{\pi_j}{\pi_i} = \frac{W_{1i}}{W_{i1}} \frac{W_{21}}{W_{12}} \cdots \frac{W_{jk}}{W_{kj}}. \tag{S3}$$

If there are multiple paths between i and j, this product of ratios will be the same for each path (this is exactly equivalent to the condition of detailed balance), so we may restrict attention to a single one. Now suppose i represents a system state with n_i ligands bound, and j represents a system state with n_j ligands bound. If along the (directed) path from i to j, p transitions represent binding of the ligand and along the (directed) reverse path from j to i, q transitions represent binding, then $p - q = n_j - n_i$.

Now we make our key assumption, which is the mass-action assumption that the transitions representing binding have rates linear in the ligand concentration x. We also assume that all other transition rates are independent of x. This means we can write

$$\pi_i = \frac{1}{1 + \sum_{j \neq i} x^{n_j - n_i} A_{ji}}, \tag{S4}$$

where A_{ji} is a positive number depending on transition rates but independent of x.

We are interested in fact in the steady-state probability of some set of states X, which we can now write as:

$$\pi_X = \sum_{i \in X} \pi_i = \sum_{i \in X} \frac{1}{1 + \sum_{j \neq i} x^{n_j - n_i} A_{ji}}. \tag{S5}$$

The logarithmic sensitivity of π_X to changes in x can now be computed by differentiating:

$$\frac{\partial \log \pi_X}{\partial \log x} = -\frac{1}{\pi_X} \sum_{i \in X} \left(\pi_i^2 \sum_{j \neq i} (n_j - n_i)\, x^{n_j - n_i} A_{ji} \right) = -\frac{1}{\pi_X} \sum_{i \in X} \left(\pi_i^2 \sum_{j} (n_j - n_i) \frac{\pi_j}{\pi_i} \right) \tag{S6}$$

$$= -\frac{1}{\pi_X} \sum_{i \in X} \pi_i \left(\sum_j (n_j - n_i)\, \pi_j \right) = -\frac{1}{\pi_X} \sum_{i \in X} \pi_i \left(\sum_{j \in X} (n_j - n_i)\, \pi_j + \sum_{j \notin X} (n_j - n_i)\, \pi_j \right), \tag{S7}$$

now we do the sums over j:

$$\frac{\partial \log \pi_X}{\partial \log x} = -\frac{1}{\pi_X} \sum_{i \in X} \pi_i \left(\pi_X \langle n_b \rangle_X - n_i \pi_X + \pi_{\overline{X}} \langle n_b \rangle_{\overline{X}} - n_i \pi_{\overline{X}} \right), \tag{S8}$$

and those over i, yielding the result, (10):

$$\frac{\partial \log \pi_X}{\partial \log x} = -\left(\pi_{\overline{X}}\langle n_b\rangle_{\overline{X}} - \pi_{\overline{X}}\langle n_b\rangle_X\right) = \left(\langle n_b\rangle_X - \langle n_b\rangle_{\overline{X}}\right)(1-\pi_X). \tag{S9}$$

What goes wrong in this argument when detailed balance is broken? In that case, the ratio π_j/π_i depends on the transition rates not according to (S3), but in a more complicated way given in general by the Markov chain tree theorem, as described in the main text. The ratio π_j/π_i is no longer a homogeneous function of x, but is a quotient of polynomials in x (see 29).

Equation (10) is closely related to the well-known theorems relating response to fluctuations at thermodynamic equilibrium (see e.g. Section 7.1 in [1]) and similar expressions have also appeared in a biophysical context (Eq. 8.2 in [2]).

Supplementary Note 2. APPLICATION OF THE EQUILIBRIUM EQUALITY (10) TO THE FINDINGS OF FUKUOKA ET AL.

In the experiment of Fukuoka et al. [3], *E. coli* cells expressing CheY-GFP fusion protein were tethered to a substrate by one of their flagellar filaments, enabling simultaneous observation of rotational direction (the whole cell body rotates) and CheY binding to the motor, which can be quantified by the intensity of a fluorescent CheY-GFP spot at the center of rotation. The authors observe spontaneous directional switching and report the average numbers of CheY-P bound to the motor when it rotates clockwise and when it rotates counterclockwise.

The spontaneous directional switching of different motors on the same cell has also been observed to be synchronized [4], which suggests that the switching may be due to spontaneous (intrinsic) fluctuations in the CheY-P concentration in the cell. It might be, then, that the numbers of bound CheY-P reported by Fukuoka et al. should be thought of as measurements at two slightly different CheY-P concentrations x_- and $x+$, with $x_- < x_+$. By contrast, the right hand side of (10) involves the difference of the number bound $\langle n_b\rangle_{\mathrm{CW}}(x) - \langle n_b\rangle_{\mathrm{CCW}}(x)$ at a single, fixed concentration x.

However, for any detailed balanced model, the mean number bound in each state $\langle n_b\rangle_{\mathrm{CW}}(x)$ and $\langle n_b\rangle_{\mathrm{CCW}}(x)$ are increasing functions of x. This means that for any $x_- < x <$

x_+,

$$\langle n_b \rangle_{\mathrm{CW}}(x) - \langle n_b \rangle_{\mathrm{CCW}}(x) < \langle n_b \rangle_{\mathrm{CW}}(x_+) - \langle n_b \rangle_{\mathrm{CCW}}(x_-), \tag{S10}$$

and that the measurement $11 = 13 - 2$ of Fukuoka et al.—the right hand side of this inequality—should exceed the effective Hill coefficient, in any detailed balanced scheme.

Supplementary Note 3. SATURATION OF THE NONEQUILIBRIUM MWC BOUND, (11)

Here we show that the bound (11) can be approached arbitrarily closely in an appropriate limit of transition rates, and that in fact, π_{CW} can be made to approach a Hill function with $H = 2n$.

Suppose that binding and unbinding of CheY-P is very fast compared to the CCW $\leftrightarrow$ CW switching transitions. Suppose also that CheY-P binds at equilibrium with dissociation constant depending on the rotation state, K_{CW} or K_{CCW}, independently (non-cooperatively) to each binding site. Finally, suppose that the CCW $\rightarrow$ CW transition can only occur when n CheY-P molecules are bound, and the CW $\rightarrow$ CCW transition can only occur when none are bound. Suppose that when they can occur, these directional switching transitions occur at rate r.

Under these assumptions, the penultimate of which breaks detailed balance, the effective rate of the CCW $\rightarrow$ CW transition is given by r times the probability of n molecules being bound, given that the rotation state is CCW, which is $x^n / (K_{\mathrm{CCW}} + x)^n$. Similarly, the effective rate of the CW $\rightarrow$ CCW transition is given by r times the probability of no molecules being bound, given that the rotation state is CW, which is $K_{\mathrm{CW}}^n / (K_{\mathrm{CW}} + x)^n$. Note that these assumptions are qualitatively similar to Tu's [5] choice of parameters—which posit a nonequilibrium enhancement of the CW $\rightarrow$ CCW transition when few CheY-P are bound and an enhancement of CCW $\rightarrow$ CW transition when many CheY-P are bound. Such an enhancement might arise from coupling of the switching transitions to the torques generated by the motor [6, 7].

Given these choices, the steady-state clockwise bias is given by

$$\pi_{\mathrm{CW}}(x) \approx \frac{\left(\frac{x}{K_{\mathrm{CCW}}+x}\right)^n}{\left(\frac{x}{K_{\mathrm{CCW}}+x}\right)^n + \left(\frac{K_{\mathrm{CW}}}{K_{\mathrm{CW}}+x}\right)^n} = \frac{1}{1 + \left(\frac{K_{\mathrm{CW}}}{K_{\mathrm{CW}}+x}\right)^n \left(\frac{K_{\mathrm{CCW}}+x}{x}\right)^n}, \tag{S11}$$

where the approximate equality relies on the timescale separation mentioned above. Careful discussion and justification of this kind of approximation can be found in, e.g. Simon and Ando (1961) [8] and Courtois (1975) [9]. In that literature, a system satisfying the kind of timescale separation assumptions described above is called *nearly completely decomposable.* See also Haken (1983), pp. 204–205 [10].

Now we take $K_{\mathrm{CCW}} = K/\epsilon$ and $K_{\mathrm{CW}} = K\epsilon$, so that

$$\pi_{\mathrm{CW}}(x) = \frac{1}{1 + \left(\frac{K\epsilon}{K\epsilon+x}\right)^n \left(\frac{K/\epsilon+x}{x}\right)^n} = \frac{1}{1 + \left(\frac{K}{x}\right)^n \left(\frac{K+x\epsilon}{x+K\epsilon}\right)^n}. \tag{S12}$$

And so if we send $\epsilon \to 0$, so that the clockwise state has a much higher affinity for CheY-P $K_{\mathrm{CW}} \ll x \ll K_{\mathrm{CCW}}$, we find that π_{CW} approaches a Hill function with $H = 2n$:

$$\pi_{\mathrm{CW}}(x) \to \frac{x^{2n}}{K^{2n} + x^{2n}}, \tag{S13}$$

which saturates (11).

We want to emphasize two points regarding the application of the support bound $H_{\mathrm{eff}} \leq 2n$ to flagellar motor switching. First, n is not the difference in the means $\langle n_b \rangle_{\mathrm{CW}} - \langle n_b \rangle_{\mathrm{CCW}}$, and we are not saying that $H_{\mathrm{eff}} \leq 2\left(\langle n_b \rangle_{\mathrm{CW}} - \langle n_b \rangle_{\mathrm{CCW}}\right)$ always—this is not true. In fact, it is possible in a nonequilibrium MWC model to see a Hill coefficient of n, even if $\langle n_b \rangle_{\mathrm{CW}} - \langle n_b \rangle_{\mathrm{CCW}} = 0$ For example, if $K_{\mathrm{CCW}} = K_{\mathrm{CW}} = K$, then (S11) becomes

$$\pi_{\mathrm{CW}}(x) \approx \frac{x^n}{K^n + x^n}, \tag{S14}$$

while the average number of ligands bound is the same in the clockwise and counterclockwise states.

Second, we note that the concordance between sensitivity measurements and the number of ligands bound is not the only desideratum for a model of flagellar motor switching, and we are not putting forward the optimizer of (11) that we describe here as *the* motor mechanism.

Supplementary Note 4. EXPONENTIAL SENSITIVITY BY NESTED HYSTERESIS

In this section, we show that the mechanism of nested hysteresis, described in the main text, can achieve a sensitivity of $2^n - 1$, saturating the support bound for unordered binding. We also show that a simple modification of this mechanism—stabilizing the fully bound and

fully unbound states by scaling the rates of their exit transitions—results in the probability of the fully bound state $\pi_{\text{all}}(x)$, viewed as a function of the ligand concentration x, approaching as closely as desired a Hill function with $H = 2^n - 1$:

$$\pi_{\text{all}}(x) \to \frac{x^{2^n-1}}{1 + x^{2^n-1}}. \tag{S15}$$

To lighten notation, we will from now on suppress functional dependence on x, e.g. writing π_{all} instead of $\pi_{\text{all}}(x)$. We will also write π_{none} for the steady-state probability that no ligands are bound.

Our arguments will only establish convergence pointwise in x, which especially for this second claim—about the approach to a Hill function—may feel unsatisfactory, since a sequence of functions converging pointwise may never look similar to their limit function. Therefore, we supplement these arguments with a *Mathematica* notebook (`https://github.com/jaowen/nested-hysteresis`) with code to explicitly construct, for any n, kinetic schemes as close as desired to the limiting case we discuss here. In the special case of $n = 3$, we also provide a computer-assisted verification of an inequality that implies uniform convergence to a Hill function with $H = 7$.

The first step in our argument is to describe a nonequilibrium mechanism to double the sensitivity of any kinetic scheme, effectively by coupling it to a new binary degree of freedom in a special way—doubling its state space. Then, by iterating this construction, we will be able to build up to nested hysteresis.

I. Sensitivity doubling

Suppose we have a kinetic scheme with graph G, whose transition rates may depend (linearly) on a parameter x, and in which we distinguish two states, which we call a and b. Define

$$\tilde{\pi}_a = \sum_{\substack{\text{spanning trees of } G \\ \text{oriented to a}}} \prod_{\text{tree edges } i \to j} W_{ji} \tag{S16}$$

to be the sum of oriented spanning tree weights given by the Markov chain tree theorem (MTT, see discussion in "Materials and Methods" of main text) for the probability of state a, prior to normalization. $\tilde{\pi}_b$ is defined analogously, but with trees rooted at state b. We also need to define another quantity, which involves spanning 2-forests of G. A spanning 2-forest

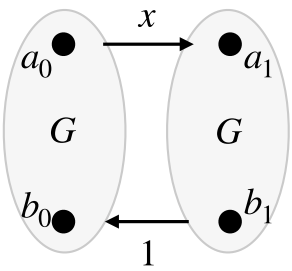

Supplementary Figure 1. **Joining two copies of G.** A new kinetic scheme constructed from two copies of G by connecting the distinguished states a' and b' with their counterparts a'' and b''.

of G is a subgraph of G with two connected components, containing every vertex, and having no cycles. The components of spanning 2-forests are trees (graphs with no cycles). If a and b are contained in different components of the spanning forest, then we can orient the forest so that one component tree is rooted at a and the other at b. We define F_{ab} to be the sum of the weights of all such forests, oriented in that way:

$$F_{ab} = \sum_{\substack{\text{spanning 2-forests of } G \\ \text{oriented to a, b}}} \prod_{\text{tree edges } i \to j} W_{ji} \tag{S17}$$

Now suppose we construct a new scheme whose graph G' is formed by joining together two copies of G as illustrated in Supplementary Figure 1, where in the copies of G we have renamed the states a and b, to distinguish each from its counterpart in the other copy of G and in the original scheme.

Our goal now will be to find, for this new kinetic scheme, the steady-state ratio $\pi_{a''}/\pi_{b'}$. We will do this by applying the MTT, starting with spanning trees rooted at a''. There are two kind of such trees. The first kind consists of two spanning trees of G (one for each copy) rooted at a' and a'', plus the edge with rate x. The second kind consists of a spanning tree of G rooted at a', a spanning 2-forest with components rooted at a'' and b'', and the edges with rates x and 1. These two classes of spanning trees of G', plus their contributions (in terms of $\tilde{\pi}_a$, F_{ab} and x) to the sum in the MTT, are illustrated in Supplementary Figure 2. The contributions are

$$\tilde{\pi}_{a''} = \tilde{\pi}_a^2 x + \tilde{\pi}_a x F_{ab} \tag{S18}$$

Reasoning similarly about the spanning trees of G' rooted at b', illustrated in Supple-

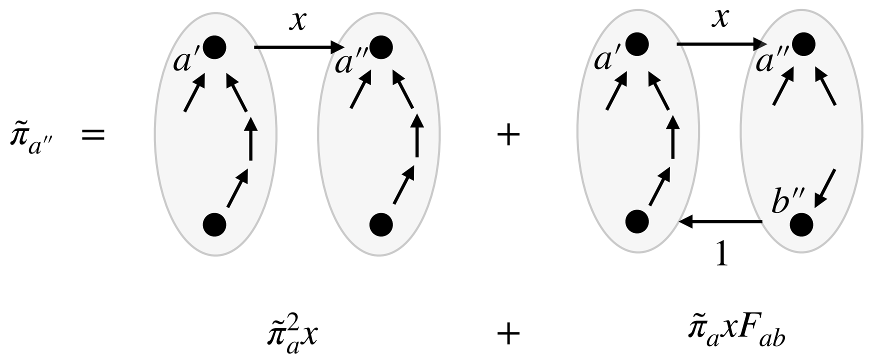

Supplementary Figure 2. **Two classes of spanning trees rooted at a''.** Spanning trees rooted at a'' fall into two classes, pictured schematically here and described in the text. Each class contributes a term—the sum of the weights of all spanning trees in the class—to $\tilde{\pi}_{a''}$.

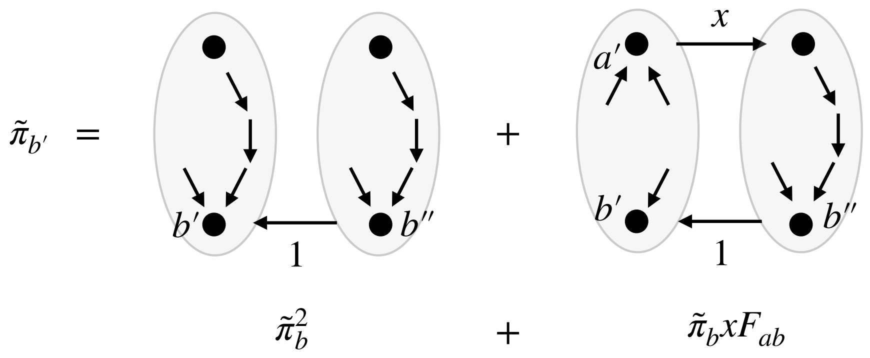

Supplementary Figure 3. **Two classes of spanning trees rooted at b'.** Spanning trees rooted at b' fall into two classes, pictured schematically here and described in the text. Each class contributes a term—the sum of the weights of all spanning trees in the class—to $\tilde{\pi}_{b'}$.

mentary Figure 3, we find the MTT sum contributions:

$$\tilde{\pi}_{b'} = \tilde{\pi}_b^2 + \tilde{\pi}_b x F_{ab} \tag{S19}$$

Putting these together, we get from the MTT that the steady-state probability ratio is given by

$$\frac{\pi_{a''}}{\pi_{b'}} = \frac{\tilde{\pi}_{a''}}{\tilde{\pi}_{b'}} = \frac{\tilde{\pi}_a^2 x + \tilde{\pi}_a x F_{ab}}{\tilde{\pi}_b^2 + \tilde{\pi}_b x F_{ab}}. \tag{S20}$$

Now we are ready to introduce the timescale separation that is critical to this mechanism. We do this by supposing that all the transition rates inside the copies of G (e.g. inside the gray ovals in the graphs of G') are scaled by the same factor s, which we will then send

towards infinity. The key fact is that the terms in (S20) are homogeneous in this scale factor s. This is because a spanning tree of a graph with N vertices always has $N-1$ edges, and a spanning 2-forest has $N-2$. So if G has N vertices, then we get

$$\frac{\pi_{a''}}{\pi_{b'}} = \frac{(s^{N-1})^2\tilde{\pi}_a^2 x + (s^{N-1})(s^{N-2})\tilde{\pi}_a x F_{ab}}{(s^{N-1})^2\tilde{\pi}_b^2 + (s^{N-1})(s^{N-2})\tilde{\pi}_b x F_{ab}} = \frac{s\tilde{\pi}_a^2 x + \tilde{\pi}_a x F_{ab}}{s\tilde{\pi}_b^2 + \tilde{\pi}_b x F_{ab}}. \tag{S21}$$

In the limit $s \to \infty$ we then get

$$\frac{\pi_{a''}}{\pi_{b'}} \to x\left(\frac{\tilde{\pi}_a}{\tilde{\pi}_b}\right)^2 = \left(\frac{\pi_a}{\pi_b}\right)^2, \tag{S22}$$

pointwise in x. Notably, it is also true that the derivative

$$\frac{\partial}{\partial x}\left(\frac{\pi_{a''}}{\pi_{b'}}\right) \to \frac{\partial}{\partial x}\left(x\left(\frac{\pi_a}{\pi_b}\right)^2\right), \tag{S23}$$

pointwise, as $s \to \infty$. This is false in general (pointwise convergence of a sequence of functions does not imply pointwise convergence of derivatives to the derivative of the limit) but is true for this rational function (S21) as a consequence of it being true for polynomials. An argument that it is true for polynomials can be found at [11]. Now to see that it is true for (S21), write $p(x,s) = \tilde{\pi}_a^2 x + \tilde{\pi}_a x F_{ab}/s$ and $q(x,s) = \tilde{\pi}_b^2 + \tilde{\pi}_b x F_{ab}/s$. These are both polynomials in x and they converge pointwise to limits $p(x) = \tilde{\pi}_a^2 x$ and $q(x) = \tilde{\pi}_b^2$, respectively, as $s \to \infty$. Now consider $\tilde{\pi}_{a''}/\tilde{\pi}_{b'} = p(x,s)/q(x,s)$. The derivative with respect to x is

$$\frac{\partial}{\partial x}\left(\frac{\tilde{\pi}_{a''}}{\tilde{\pi}_{b'}}\right) = \frac{(\partial_x p(x,s))\, q(x,s) - p(x,s)\,(\partial_x q(x,s))}{q(x,s)^2}. \tag{S24}$$

Note that as $s \to \infty$, the derivatives $\partial_x p(x,s) \to \partial_x p(x)$ and $\partial_x q(x,s) \to \partial_x q(x)$ (because p and q are polynomials in x, [11]), and so the right hand side of (S24) converges pointwise to the derivative with respect to x of $p(x)/q(x) = \tilde{\pi}_a^2 x/\tilde{\pi}_b^2$, as desired.

These arguments apply equally well to the logarithmic derivative, and so the construction described in this section can be thought of as doubling (and then adding one to) a logarithmic sensitivity. To see this, suppose for the original graph G we have $\pi_a/\pi_b = x^m$ for some m, so that $\partial \log(\pi_a/\pi_b)/\partial \log x = m$. Then the arguments above show that, in the new scheme, $\partial \log(\pi_{a''}/\pi_{b'})/\partial \log x \to 2m+1$ as $s \to \infty$. Note that the recurrence $m_1 = 1$, $m_{i+1} = 2m_i + 1$ has solution $m_n = 2^n - 1$.

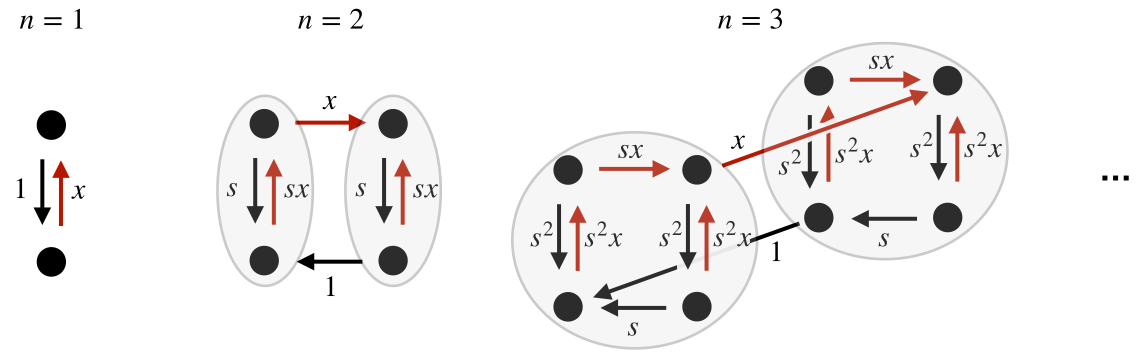

Supplementary Figure 4. **Iterative construction of the kinetic scheme of nested hysteresis.** Figure 3(a) of the main text, reproduced here for ease of reference. In each diagram, the gray ovals indicate the subsystems (corresponding to binding and unbinding to the first $n-1$ sites), which are assumed to relax much faster than the other transitions.

II. Iterative construction

We can repeat the whole construction described in the previous section, starting with the graph G' instead of G, and taking $a = a''$, $b = b'$. The iteration of this procedure—starting from a kinetic scheme with two states representing binding and unbinding of a ligand to a single site—is what is depicted in Figure 3(a) of the main text, reproduced here as Supplementary Figure 4.

To be explicit, let's denote each possible binding state by a string of zeros and ones indicating which sites are occupied. For example, when there is a single binding site $n = 1$, the two states are '0' (unoccupied) and '1' (occupied). When there are two binding sites, the states are '00', '10', '01', and '11', and so on for higher numbers of binding sites. In the case $n = 1$ the states a and b (for the purposes of the sensitivity doubling described in the last section) are $a = 0$ and $b = 1$. When constructing $n = 2$, we have $a'' = 11$ and $b' = 00$ and these become the new a and b for the next step, and so on.

In the base case ($n = 1$), the ratio of probabilities $\pi_a/\pi_b = \pi_1/\pi_0$ equals x. Then, one step of sensitivity doubling constructs a scheme (the case $n = 2$) where the ratio of two state probabilities $\pi_{11}/\pi_{00} \to x^3$ as $s \to \infty$ (pointwise). Let's define $f(x, s) \equiv \pi_{11}/\pi_{00}$.

For any finite value of s, $f(x, s)$ won't quite be x^3—does this undermine our ability to "nest" the argument and build up to a scheme which approaches x^{2^n-1} as $s \to \infty$? It turns out that it does not. To see this, let's be very explicit, considering the next step

of the iteration. We must allow that the new scale factor introduced might not take the same value as the one (called s) in the first step, so we will call the new one s'. Write $g(x,s,s') \equiv \pi_{111}/\pi_{000}$. Now, our sensitivity doubling arguments give $\lim_{s\to\infty} f(x,s) = x^3$ and $\lim_{s'\to\infty} g(x,s,s') = xf(x,s)^2$. It turns out this does imply that $\lim_{s\to\infty} g(x,s,s) = x(x^3)^2 = x^7$. To see this, note that for any ϵ there is a number S_1 such that $s > S_1$ guarantees $|xf(x,s)^2 - x^7| \le \epsilon/2$ (by the first limit and continuity of xf^2 in f), and another number S_2 such that $s' > S_2$ guarantees $|g(x,s,s') - xf(x,s)^2| \le \epsilon/2$. And so for any ϵ, choosing $s > \max(S_1, S_2)$ gives $|g(x,s,s) - x^7| \le \epsilon$. Note that S_1 and S_2 may depend on x.

To summarize, the arguments we have given so far allow us to construct, for any n, a scheme of ligand binding with rates depending on a parameter s such that

$$\frac{\pi_{\text{all}}}{\pi_{\text{none}}} \to x^{2^n - 1} \tag{S25}$$

as $s \to \infty$.

III. Stabilizing extreme states to get a Hill function

Finally, we will show here how, given a kinetic scheme with any given value for the steady-state ratio of two state probabilities $\pi_i/\pi_j = \alpha > 0$, we can create a scheme with π_i as close as desired to $\alpha/(1+\alpha)$. Importantly, the construction does not depend on the value of α.

We begin by defining, for any k,

$$\tilde{\pi}_k = \sum_{\substack{\text{spanning trees of } G \\ \text{oriented to k}}} \prod_{\text{tree edges } i \to j} W_{ji} \tag{S26}$$

to be the sum of spanning tree contributions established by the MTT to be proportional to the steady-state probability π_k. Note that we have $\tilde{\pi}_i/\tilde{\pi}_j = \pi_i/\pi_j = \alpha$, and

$$\pi_i = \frac{\tilde{\pi}_i}{\sum_k \tilde{\pi}_k} = \frac{\tilde{\pi}_i}{\tilde{\pi}_i + \tilde{\pi}_j + \sum_{k\neq i,j} \tilde{\pi}_k}. \tag{S27}$$

Now suppose we scale all the rates of transitions leaving i and j by a factor q, pictured schematically in Supplementary Figure 5, where the states i and j are labeled in cyan.

Any spanning tree oriented to a state other than i and j must include exactly one directed edge leaving i and one leaving j, which means two factors of q. Those oriented to i or j, by contrast, pick up only one factor of q (for leaving j, or i, respectively). This means that

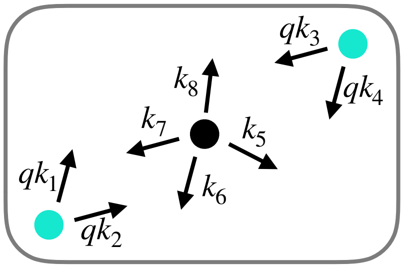

Supplementary Figure 5. **Stabilizing extremes.** Scaling the rates leaving two states (cyan disks) by a small quantity q yields a version of nested hysteresis that (in an appropriate limit) yields a Hill function with $H = 2^n - 1$.

scaling by q changes (S27) to

$$\pi_i = \frac{q\tilde{\pi}_i}{q\tilde{\pi}_i + q\tilde{\pi}_j + q^2 \sum_{k\neq i,j} \tilde{\pi}_k} = \frac{\tilde{\pi}_i}{\tilde{\pi}_i + \tilde{\pi}_j + q \sum_{k\neq i,j} \tilde{\pi}_k}. \tag{S28}$$

And so, in the limit $q \to 0$, we find

$$\pi_i \to \frac{\tilde{\pi}_i}{\tilde{\pi}_i + \tilde{\pi}_j} = \frac{\alpha}{1+\alpha}, \tag{S29}$$

as desired.

Applying this "stabilization of extremes" to nested hysteresis finally gives

$$\lim_{q\to 0} \lim_{s\to\infty} \pi_{\text{all}} = \lim_{q\to 0} \frac{x^{2^n-1}}{1 + x^{2^n-1} + q\left(\sum_{j=1}^{2^n-2} x^j\right)} = \frac{x^{2^n-1}}{1+x^{2^n-1}}. \tag{S30}$$

IV. The cases $n = 2$ and $n = 3$

For illustrative purposes, we give here explicit expressions for the probability of the fully bound state π_{all}, in nested hysteresis with stabilized extremes, in the cases $n = 2$ and $n = 3$. We choose $q = 1/s$, but leave s as a parameter. It turns out that this choice of q allows us to describe (for $n = 2, 3$) the limiting procedure leading a Hill function with $H = 2^n - 1$ as a single limit $s \to \infty$. Note that we do not prove that this choice of s dependence for q works for any n. Instead our arguments above only establish that the iterated limit, first of $s \to \infty$ and then of $q \to 0$, leads to a Hill function with $H = 2^n - 1$.

$$\pi_{\text{all}} = \pi_{11} = \frac{x^2(sx+1)}{s\left(x^3+1\right) + 2x(x+1)} \tag{S31}$$

$$\pi_{\text{all}} = \pi_{111} = \frac{x^3(sx+1)^2\left(s^2x^2+s+x\right)}{s^4(x^7+1)+s^3x(x+1)(x^2+x+1)(x(3x-4)+3)+s^2x(x+1)(x^2+x+1)^2+3sx^2(x+1)(x^2+1)+2x^3(x+1)} \quad \text{(S32)}$$

The code used to generate these expressions can be found in the supplemental *Mathematica* notebook, available online at `https://github.com/jaowen/nested-hysteresis`. The same code can be used to generate explicit expressions at least up to $n = 5$—they become very large but *Mathematica* confirms the correct limit as $s \to \infty$, e.g. in the case $n = 5$ the limit being $x^{31}/(1 + x^{31})$.

SUPPLEMENTARY REFERENCES

[1] Robert Zwanzig. *Nonequilibrium statistical mechanics.* Oxford University Press, 2001.

[2] Jeffries Wyman Jr. Linked functions and reciprocal effects in hemoglobin: a second look. *Advances in Protein Chemistry*, 19:223–286, 1964.

[3] Hajime Fukuoka, Takashi Sagawa, Yuichi Inoue, Hiroto Takahashi, and Akihiko Ishijima. Direct imaging of intracellular signaling components that regulate bacterial chemotaxis. *Science Signaling*, 7(319):ra32–ra32, April 2014. ISSN 1945-0877, 1937-9145. doi: 10.1126/scisignal.2004963. URL `https://stke.sciencemag.org/content/7/319/ra32`.

[4] Shun Terasawa, Hajime Fukuoka, Yuichi Inoue, Takashi Sagawa, Hiroto Takahashi, and Akihiko Ishijima. Coordinated reversal of flagellar motors on a single Escherichia coli cell. *Biophysical journal*, 100(9):2193–2200, 2011.

[5] Yuhai Tu. The nonequilibrium mechanism for ultrasensitivity in a biological switch: Sensing by Maxwell's demons. *Proceedings of the National Academy of Sciences*, 105(33):11737–11741, August 2008. ISSN 0027-8424, 1091-6490. doi:10.1073/pnas.0804641105. URL `https://www.pnas.org/content/105/33/11737`.

[6] Fangbin Wang, Hui Shi, Rui He, Renjie Wang, Rongjing Zhang, and Junhua Yuan. Nonequilibrium effect in the allosteric regulation of the bacterial flagellar switch. *Nature Physics*, 13(7):710–714, July 2017. ISSN 1745-2481. doi:10.1038/nphys4081. URL `https://www.nature.com/articles/nphys4081`.

[7] Bin Wang, Yuhui Niu, Rongjing Zhang, and Junhua Yuan. Dynamics of switching at stall reveals nonequilibrium mechanism in the allosteric regulation of the bacterial flagellar switch.

bioRxiv, 2021.

[8] Herbert A Simon and Albert Ando. Aggregation of variables in dynamic systems. *Econometrica: Journal of the Econometric Society*, pages 111–138, 1961.

[9] Pierre-Jacques Courtois. Error analysis in nearly-completely decomposable stochastic systems. *Econometrica: Journal of the Econometric Society*, pages 691–709, 1975.

[10] Hermann Haken. Synergetics: An Introduction. Nonequilibrium Phase Transitions and Self-Organization in Physics, Chemistry and Biology. *New York: Springer-Verlag*, 1983.

[11] Joey Zou (https://math.stackexchange.com/users/260918/joey zou). Derivative of a limit of a sequence of polynomials equal to the limit of derivatives? Mathematics Stack Exchange. URL `https://math.stackexchange.com/questions/1959689/derivative-of-a-limit-of-a-sequence-of-polynomials-equal-to-the-limit-of-derivat/1959915`.
